# The effect of training problem-solving skills for pregnant women experiencing intimate partner violence: a randomized control trial

**DOI:** 10.11604/pamj.2018.30.79.14872

**Published:** 2018-05-29

**Authors:** Ziba Taghizadeh, Maryam Pourbakhtiar, Sogand Ghasemzadeh, Khadijeh Azimi, Abbas Mehran

**Affiliations:** 1 Nursing and Midwifery Care Research Center, Tehran University of Medical Sciences, Tehran, Iran; 2 School of Nursing and Midwifery, Tehran University of Medical Sciences, Iran; 3 Department of Psychology and Education of Exceptional Children, University of Tehran, Tehran, Iran; 4 Master of Biostatistics, Faculty of Tehran University of Medical Sciences, Tehran, Iran

**Keywords:** Violence against women, types of intimate partner violence, problem-solving skills, pregnant women

## Abstract

**Introduction:**

Intimate partner violence (IPV) in pregnancy is considered as an additional threat to the maternal/fetal health. The present study was aimed to investigate the effectiveness of training problem-solving skills on IPV against pregnant women.

**Methods:**

The present randomized clinical trial was conducted on 125 and 132 women visiting the health centers of Tehran as the intervention and the control groups, respectively; samples were selected using random stratified cluster sampling. The intervention group underwent four problem-solving training sessions. Three months later, both groups completed the revised Conflict Tactics Scale questionnaire. Data were analyzed using SPSS v.16.

**Results:**

The mean (SD) ages of the participants were 27.51 (4.26) and 27.02 (4.26) years, respectively, in the control and the intervention groups. The rates of the physical and psychological violence were significantly reduced after the intervention in the intervention group. Risk differences of the physical, psychological and sexual violence before and after the intervention were 3% (95% CI: -8.23 to14.13, P = 0.6), 1.5% (95% CI: -4.93 to 8.03, P = 0.6) and 4.8% (95% CI: -7.11 to 16.52, P = 0.4) in the control group and 8.8% (95% CI: -3.47 to 20.71, P = 0.1), 25.4% (95% CI: 15.77 to 34.66, P < 0.001) and 4.9% (95% CI: -7.38 to16.97, P = 0.4) in the intervention group, respectively.

**Conclusion:**

It seems that training this skill as a part of the routine prenatal care could be effective in reducing intimate partner violence.

## Introduction

Intimate partner violence (IPV) is an act of physical, sexual and emotional-psychological abuse by the partner and is considered a health problem throughout the world [1]. According to the World Health Organization (WHO), physical violence may take place in the form of slapping, throwing objects, pushing, punching, kicking or using any weapons to harm the other person; sexual violence involves engaging in any sexual act through force or out of fear and any act that humiliates the woman; emotional violence involves the acts of disrespect, insult, disdain, isolation from the family and friends, controlling decision-making etc [2]. Although physical violence is more easily recognized due to its battering signs, psychological and emotional violence are substantially more harmful [3]. Recent studies by the WHO and the demographic health surveys (DHS) conducted in different countries indicated a varying prevalence of domestic violence against women by their intimate partners, ranging from 15% to 75% in a lifetime [4]. A nationwide survey carried out in 28 province centers of Iran showed that 30% of married women have at least experienced one type of intimate partner violence in their lifetime [5]. Due to its violation of human rights and the great effects on the community's health and development, intimate partner violence has become a major global concern [6]. In some studies, pregnant women are recognized as one of the most vulnerable groups of victims of this violence [7] and the most common type of violence is, in fact, intimate partner violence against pregnant women [8]. In a systematic global review, the prevalence of intimate partner violence against pregnant women was reported as 1% to 20% [6], reaching 15% to 71% in low to middle-income countries [9] and 4% to 48% in Asian countries [6]. In Iran, the mean prevalence of IPV against pregnant women was reported as more than 60% [10]. In a study conducted in Tehran province, 60.6% of pregnant women had experienced different types of IPV, with 60.5% reporting psychological, 23.5% sexual and 14.6% physical violence [5]. For many reasons, the reported statistics are lower than the actual figures [11]. The type of committed intimate partner violence is different for women who are experiencing it for the first time during their pregnancy. A study by Martin et al (2004) showed a relationship between pregnancy and increased psychological and sexual violence, even in women with no experience of violence before pregnancy. A number of studies have shown that emotional-psychological violence is highly likely to continue into pregnancy in women with the experience of IPV before their pregnancy [12].

The complications caused by IPV during pregnancy are associated with the type of experienced violence. There is a significant relationship between verbal abuse and low birth weight. Sexual violence during pregnancy can also lead to placental abruption, miscarriage and preterm labor [13], while physical violence during pregnancy is associated with direct effects on the fetus, including fetal bone fractures or fetal death [5]. Irrespective of the changes in the type of the committed IPV against pregnant women, pregnancy is, in fact, the best time for performing interventions on abused women [14]. During pregnancy, intimate partner violence is more prevalent than other common complications such as preeclampsia and gestational diabetes [15], which are among the main causes of maternal mortality [12] and for which women are routinely examined [16]. In fact, pregnancy is a high-risk period for abused women, when the health of both the mother and the fetus is at risk [17]; and when the health is even further threatened with the adoption of inappropriate coping strategies against intimate partner violence [18]. Abused pregnant women use various coping strategies to deal with intimate partner violence. In a study conducted by Zakar et al (2012), most abused pregnant women in Pakistan used coping strategies such as avoiding their spouse, participating in religious ceremonies and events, joining local support networks, asking for help from the family and friends or reticence, which, although have been helpful in reducing the physical violence, had no effect on psychological violence [19]. Problem-oriented coping is a coping strategy adopted in stressful conditions that focuses on the existing problem, plans for solving it and pursues help in others [20]. The problem-oriented coping strategy is closely connected to problem-solving skills. Problem-solving skills are therefore crucial life skills that everybody should possess [21]. Problem-solving skills are a great help to doctors, who often use it in therapy sessions. The assumption is that successful problem solving reduces people's incompatibility and leads to positive compatibility in a life filled with routine problems [22]. In one study, however, teaching to forgive one's spouse was reported to be a more effective strategy than life skills training (including problem-solving skills) in improving symptoms of depression and anxiety in abused women [23].

Health centers are frequented more often by women of reproductive age and pregnant women who seek medical care [24]. Various studies have shown that, for many different reasons, pregnancy [15] is the best time for performing interventions in health centers in order to reduce and prevent the risk of IPV against them; the first reason is that 95% of pregnant women visit the health centers during pregnancy to receive routine pregnancy care [25]; second, due to the changes taking place in the woman's role (i.e, the adoption of a maternal role) and her wishes to protect the fetus and herself, pregnancy is a suitable period for training behavior changes; and third, regular visits to the health center during pregnancy could lead to an empathic and trusting relationship between the mother and the midwife [15]. It has been witnessed during the recent decades that countries have prioritized identifying abused women and performing primary measures for prevention and management of intimate partner violence for women's health, and a variety of educational, supportive and legal interventions have been performed for proper dealing with women's main health problems [26]. In the health centers of Iran, no specific services are currently provided for abused women during the routine pregnancy cares. The high prevalence and the negative consequences of IPV against pregnant women provide a golden opportunity for performing interventions. The lack of sufficient studies in this area shows the need for investigating the effectiveness of training problem-solving skills in changing the type of IPV against pregnant women. So the researchers decided to study the effect of problem-solving education on the prevalence of each type of domestic violence among pregnant women.

## Methods

The present quasi-experimental study was conducted in Tehran Province over a period of eight months on samples selected through random stratified cluster sampling. The researcher randomly selected two health networks affiliated with Tehran University of Medical Sciences (lot drawing) in Shahr-e-Rey and southern Tehran, as the intervention and the control groups, respectively. Then eight centers from each of the southern Tehran network and Shahr-e-Rey network were randomly selected (using the table of random numbers). The assigned centers to the intervention group and the control groups had a good dispersion and were not affiliated with each other to prevent participants from running into each other. The inclusion criteria were being pregnant in the first half of their pregnancy, having reading and writing literacy and having no previous participation in similar studies. The exclusion criteria were occurrence of a stressful event such as death and serious illness of a family member, unwillingness to cooperate, and missing more than one session. Eligible pregnant women were contacted and briefed on the study objectives and invited to participate in the study by visiting the health center. The revised Conflict Tactics Scale (CTS2) scale was completed by all the pregnant women that attended. All the participated women were fluent in Persian. Explanations on how to complete the scale were provided by the midwife (researcher). After carrying out the violence screening, 284 eligible abused women were remained and invited to take part in an orientation session. The orientation session began with the researcher introducing herself and the objectives of her study and continued with the abused pregnant women submitting their informed consents for participation. Then 142 women were randomly assigned to two groups of control and intervention. After completing the demographic details and the obstetrics history forms, the control group was requested to visit the center three months later for completing the violence screening form. In this study, IPV was assessed using CTS2 (developed by Strauss, Hamby, Boney-Mccoy, and Sugarman, 1996), which concurrently assesses the prevalence, type, and severity of the violence. The original version of CTS2 contains 78 items, which was translated into Farsi and validated by Behboudi et al (2010), who found an internal consistency of 0.8 as the reliability of the scale [27]. The revised scale included 18, 7 and 5 items in the physical, psychological and sexual dimensions respectively. Items 1 to 7 assess couple's agreement or disagreement on resolving conflicts (given the study objectives, items 7 to 36 were used only). This scale has two methods for scoring; Likert scoring or Dichotomized Scores. Dichotomized scores are used in the calculation of rates [28]; that is used in this study.

Four problem-solving skills training sessions were scheduled with the intervention group and according to their preferences (one session per week, lasting 90 minutes) (Table 1). Training was held in the form of groups by a trained midwife (researcher) with qualification in problem-solving teaching. To maintain the safety of the abused women and the midwife (researcher) from the aggression of the pregnant women's husbands in the course of the study, training was advertised only as “training problem-solving skills for pregnant women.” Participants were asked not to share their training with others until the end of the study. Meanwhile, women of both groups that needed counseling or help were confidentially referred to the relevant organizations. The used techniques in the training sessions included class activity, role play and home assignments. Each new session began with an overview of the materials taught in the previous session and an assessment of the home assignments and ended with a Q&A to resolve all the queries and ambiguities. At the end of the fourth session, participants were asked to visit the health centers two months later to complete the violence screening questionnaire again. This study was approved by the ethics committee of Tehran University of Medical Sciences and registered in Iranian Registry of Clinical Trial (Code: IRCT2013041713046N1). The confidentiality of the participants' data was ensured by allocating a separate room in each health center to the research to enable holding a private session between the midwife (researcher) and each participant. Participants in the control and the intervention groups referred to receive support services at the end of the study. The researchers asked the question of “Whether you referred to receive support services” at the beginning of each session, to control the effect of receiving supportive services. For calculating the prevalence of each type of violence, first, the score of each type of violence was divided into two states of zero and more than one; zero meant not having experienced violence and more than one meant, having experienced violence during the past year. Accordingly, the rate of violence was measured and compared before and after the intervention in both groups via risk difference and relative risk. All the analyses were performed using SPSS V.14.

**Table 1:** The content of the problem-solving training sessions

Session	Detail
1	General briefing on the problems occurring in a marriage, coping with problems (problem-oriented and emotion-oriented coping strategies) and introduction to the components of problem-solving skills.
2	The first and second stages of problem-solving skills training (accepting and carefully defining the problem).
3	The third and fourth stages of problem-solving skills training (brainstorming and solution assessment).
4	The fifth and sixth stages of problem-solving skills training (implementing the best solution and a review of the chosen solutions).

## Results

**Figure 1:**
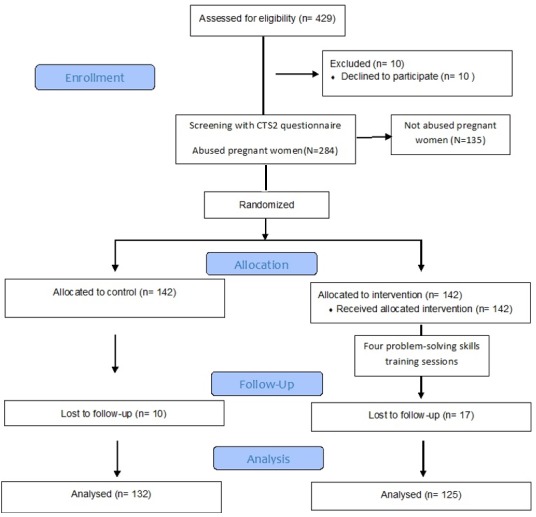
Sampling method and random allocation of the mothers to the intervention and the control groups

By the end of the study, only 132 women were left in the control group (a sample loss of 10) and 125 others in the intervention group (a sample loss of 17). Sample loss was occurred for different reasons, including not visiting the center for the second time as advised (n = 5), concerns about answering the questions on violence (n = 5), failure to attend all the sessions of training (n = 10) or immigration to other regions (n = 7) (Figure 1). The mean and standard deviation of age in the control group was 27.51 ± 4.26 and in the intervention group was 27.02 ± 4.26. Pregnancy was planned and wanted in 70% of the control group and 66% of the intervention group. Other demographic details are shown in Table 2. According to Table 3, there were no significant differences between the types of experienced IPV before the intervention, in the two groups. But, results of relative risk showed that the rate of physical and Psychological violence was significantly reduced after the intervention in the intervention group. Risk differences of physical, Psychological and sexual violence before and after the intervention were 3% (95% CI: -8.23 to14.13, P = 0.6), 1.5% (95% CI: -4.93 to 8.03, P = 0.6) and 4.8% (95% CI: -7.11 to 16.52, P = 0.4) in the control group and 8.8% (95% CI: -3.47 to 20.71, P = 0.1), 25.4% (95% CI: 15.77 to 34.66, P < 0.001) and 4.9% (95% CI: -7.38 to16.97, P = 0.4) in the intervention group.

**Table 2:** Demographic characteristics of the control and the intervention groups separately

Demographic Characteristics of Pregnant Women	Control (n=132)	Intervention (n=125)	p-value
	N (%)	N (%)	
**Education**			
Under-diploma	56 (42.5)	48(38.4)	0.9
Diploma	42(31.8)	45(36)	
College degree	11(8.3)	8(6.4)	
**Occupation**			
Housewife	124(93.9)	123(98.4)	0.2
Employee	8(6.1)	2(1.6)	
**Place of residence**			
Separate House	96(72.8)	99(79.2)	0.4
With her family	4(3)	2(1.6)	
With spouse’s family	32(24.3)	24(29.2)	
**Economic Situation**			
Good	22(17.7)	20(15.2)	0.8
Partially good	72(58)	75(63.6)	
bad	30(24.3)	25(21.2)	
**History of Past Marriages**	3(3.3)	5(4)	0.4
**Having children from Past Marriages**	1(0.8)	2(0.9)	0.99
**Number of previous children**			
0	45(34.1)	48(38.4)	0.9
1	53(40.2)	44(23.5)	
2	27(20.4)	26(20.8)	
≥3	7(5.3)	7(5.6)	

**Table 3:** Frequency of the types of intimate partner violence before and after the intervention in the intervention and the control groups

Types of intimate partner violence		Control	Intervention	Relative risk	95% CI	p
physical violence	Before	68.9%	60 %	0.87	0.72 to 1.04	0.14
	After	65.9%	51.2%	0.78	0.63 to 0.93	0.01
Psychological violence	Before	93.9%	92.8 %	1.01	0.94 to 1.08	0.7
	After	92.4 %	67.4 %	0.73	0.64 to 0.83	<0.001
sexual violence	Before	52.8%	55.3 %	1.17	0.93 to 1.47	0.1
	After	57.6 %	50.4 %	0.87	0.69 to 1.09	0.25

## Discussion

Researchers have not reached a consensus regarding the prevalence of IPV during pregnancy [24]. Pregnancy can lead to changes in the overall prevalence of intimate partner violence [29] and is thus the best time for assessing the prevalence of IPV. Various studies have shown that women with a history of committed violence against them have no desire to disclose their experiences; however, due to their frequent visits to pregnancy care centers to receive routine pregnancy care (almost 90%) and their ongoing relationships with the health service providers [16], particularly with the midwives, pregnancy provides a window of opportunity for assessing the prevalence of IPV against women [14]. In concordance with the results of the present study, the prevalence of intimate partner violence against pregnant women visiting the health centers in Tehran was reported as 88.3% [30] and 67.8% [31]. In contrast, Hassanzadeh et al (2011) reported the prevalence of intimate partner violence against pregnant women visiting the health centers in the city of Ahwaz as 19.3% [32]. This result is inconsistent with the findings of the present study. The disparity between the results obtained by the present study and other studies might be partly due to the differences in the assumed definition of IPV, the instances of IPV, the cultural and social settings of the examined societies (cultural differences refer to the tendency of women from different countries to disclose their experiences of intimate partner violence), the geographical environment, the study methodology, the used data collection tools (absence of a standard tool compatible with the common types of committed violence against women in the country), the personal attributes of participants (such as age group) and the study settings. According to Table 3, physical violence against pregnant women was reported to have occurred in 60% of the intervention group and 68.9% of the control group. A study conducted by Tivari et al (2005) in Hong Kong reported the prevalence of physical violence against pregnant women as 42% [33] and another study by Humphries et al (2012) examining 50 pregnant women in California reported this prevalence to be 48%, which is consistent with the findings of the present study [15].

In another study, Khadivzadeh et al (2011) reported that 16.5% of pregnant women in Mashhad have been subjected to physical violence [16], which is inconsistent with the findings of the present study, perhaps due to the cited study's smaller sample size and the different type of questionnaire that was used to examine the participants. In the present study, the prevalence of psychological violence against pregnant women was reported as 92.8% in the intervention group and 94% in the control group. Taherkhani et al (2009) reported the prevalence of psychological violence against women visiting the health centers as 87.3% and Faramarzi et al (2005) reported the prevalence of women visiting the health centers and obstetrics and midwifery clinics in Babol as 81.5% [34], which is consistent with the results of the present study; however, Tivari et al (2005) reported the prevalence of psychological violence against pregnant women visiting the health centers across Hong Kong to receive routine pregnancy care as 58%, which is inconsistent with the results of the present study. The majority of studies that was conducted in Iran have shown that the prevalence of psychological violence is higher than other types of IPV in pregnant women, which may be due to the greater efforts made by husbands to support pregnant women and to eliminate other types of violence, including physical violence, which make the offender resort to psychological violence. In the present study, the prevalence of sexual violence was reported as 52.8% in the intervention group and as 55.3% in the control group. Faramarzi et al (2005) reported the prevalence of sexual violence against pregnant women visiting the health centers and obstetrics and midwifery clinics in Babol as 42.2%; Avidgoic et al. reported the prevalence of sexual violence as 43.5% in Bosnia and Herzegovina; and Hesami et al (2010) reported the prevalence of this type of violence as 55.1%; these findings are consistent with the results of the present study. In contrast, Dowlatian et al (2008) reported the prevalence of this type of violence as 89.2% in Marivan [35]. The disparity of findings may be due to the fact that the examined pregnant women in the cited study were in their last month of pregnancy, which, due to the changes occurring in the women's physical appearance and her reduced tendency to engage in sexual activities, becomes a period highly prone to sexual violence; the increased sexual violence reports during this critical time in pregnancy is therefore not unexpected. Physical violence thus appears to be the least common type of reported IPV during pregnancy, which may be due to the dominant cultural and religious beliefs of the country, the husbands' greater efforts during this critical period to take care of their wives and also that women would avoid the acts that trigger their husbands to commit physical violence against them in an effort to protect their fetus and cause less harm to it.

The results of the present study showed a significant difference in the type of the physical and psychological IPV after the intervention. The intervention has thus only been able to reduce the physical and psychological types of committed violence against pregnant women. No significant differences were observed in the sexual type of committed violence against pregnant women, and the intervention did not manage to reduce the incidence of this particular type of violence. Tivari et al (2005) found a significant reduction in the psychological type of violence against 51 examined pregnant women in Hong Kong after the intervention, but no significant differences in the sexual type of violence against them. Kelly et al (2011) compared the physical and sexual types of violence against 521 pregnant women in California and found a significant reduction in the physical type of violence against the intervention group compared to the control group in the first follow-up of the intervention and then 4-8 weeks after childbirth [36], but no significant changes in the sexual type of violence during pregnancy or after childbirth were observed, which is consistent with the results of the present study. In contrast, Parker et al (1999) found a significant reduction in both physical and non-physical (i.e, psychological and sexual) violence against 132 pregnant women visiting the health clinics in Texas and Virginia in every follow-up of their intervention [37], which is inconsistent with the results of the present study; nevertheless, no significant differences were observed 6 and 12 months after the intervention, which is inconsistent with the results of the present study. According to the researcher, the disparity of the results may be due to the type of the used questionnaire in the cited study (the ASS), its follow-up period (6 and 12 months after the intervention), its intervention design (the empowerment of abused women in three sessions) and the performed intervention in the control group (which was provided through brochures containing information on sources of support during violence). The problem-solving ability is the same as the previous experience of dealing with problems, determining the barriers to solving the problem, and motivating for behavior change. The behavior of solving the problem is associated with personal attributes and thus people with problem-solving skills are more successful in dealing with problematic situations.

## Conclusion

It seems that teaching problem-solving skills can reduce these psychological types of intimate partner violence against pregnant women and their complications, since through this skill, pregnant women can learn to choose the best way out of all possible ways for dealing with their husband's physical and psychological violence (e.g, behavior change at the time of violence). Other skills such as assertiveness (being able to say no to inappropriate sexual demands by men) may be helpful in reducing the sexual type of violence against women. In general, irrespective of the type of the committed violence against pregnant women, pregnancy provides the best opportunity for performing interventions on women. So, it is recommended that life skills training classes (including material on problem-solving skills) should be held by midwives for abused pregnant women in health centers to reduce the prevalence of intimate partner violence against this group of vulnerable individuals.

**Study limitations**: Different characteristics of people in expressing emotions and personal problems, especially violence, and also women's fear of disclosure of secrets, and their husband's violence (for various reasons) may have diminished accuracy and rigor of this study. To solve this problem, through counseling skills, the researcher was able to create the opportunity for the pregnant women to express emotions, ask questions and talk about intimate partner violence. Also, some unavoidable counterfactuals might have attributed to the changes in violence victimization, e.g, cross-over, not accounting for prior victimization.

### What is known about this topic

- The prevalence of IPV against pregnant women is high;
- IPV can cause serious complications in pregnancy;
- Pregnancy is the best time for interventions in order to reduce and prevent the risk of IPV.

### What this study adds

- Teaching problem-solving skills can reduce physical and emotional types of IPV against pregnant women and their complications.
